# Discoloration of Gold Plugs

**Published:** 1877-06

**Authors:** 


### ARTICLE VI.

### *Discoloration of Gold Plugs.*

After having devoted much time and exercised the highest skill for the production of gold fillings that would, for years, stand as monuments of glory to the progressive spirit of dentistry, it is certainly mortifying, not to say discouraging, to have these same gold plugs present themselves in a few months with a dark and sometimes coppery discoloration, covering completely the exposed surface.

Prof. Chandler, of Boston, has been investigating the subject and has recently written an interesting article, in which he details experiments in the manipulation of gold foil previous to its introduction into the cavity. After subjecting gold to a great variety of tests he comes to the onclusion that the discoloration is due to the minute particles of steel that are constantly worn off from the points of the pluggers, and become incorporated in the mass of the gold plug ; this steel, he thinks, then becomes oxidised and gives rise to the discoloration. This theory of Prof. Chandler would undoubtedly do very well if it could be shown that sufficient iron (steel) was worn from the serration of a plugger to produce the results complained of; but unfortunately this cannot be done. I have a set of thirteen pluggers which I have used constantly for several years; the total loss of weight sustained by these instruments during this period (they have never been re sharpened) is two grains. Now, during this time, this set of pluggers have condensed no less than twenty ounces of gold foil, or in other words two grains of iron (or steel) have become incorporated with twenty ounces of gold, which is one-tenth of a grain to the ounce, or about the one thousandth part of a grain of iron in an average gold filling; and when we come to consider that but a very small portion of this one-thousandth of a grain is really on the surface of the plug (the portion discolored) it is impossible for human credulity to go to the extent of claiming that this infinitessimal particle of iron could possibly produce such effects as we often see. I am aware that the smallest conceivable portion of many metals can be detected by the flame test; thus the one millionth part of a grain of sodium imparts a yellow color to the flame. But because the presence of iron can be detected in gold which has been condensed by steel instruments, it does not argue that its presence there exists in sufficient quantity to cause a discoloration on the surface of a gold plug of such proportion as to become an unpleasant sight to every eye. While the profession is undoubtedly under many obligations to Professor Chandler for his efforts in this direction, it is evident, to me at least that we must look to other sources than the attrition of steel pluggers for this disfiguration of gold plugs. It must first be determined whether this discoloration is of an organic or inorganic character. It has always been assumed by writers that it belonged to the latter class of compounds. Yet it is possible that it is a deposit of organic coloring matter, either from food that is taken into the mouth or some ingredient of the saliva. But for the sake of argument, and in view of the fact that there is no positive evidence to the contrary, we may assume that the discoloration of gold plugs is due to the presence of some metal such as iron, lead, copper or silver. Undoubtedly most of the gold prepared for the dentist contains traces at least of some of the above metals (whether sufficient to produce this discoloration or not is the question, yet a much larger quantity in all cases than could possibly come from the *wear* of a plugger point.) Assuming again that foils do sometimes contain traces of copper and silver (the metals *most* likely to exist in gold) in sufficient quantities to produce this discoloration, it is easy to see that the sulphides and oxides would be formed and the bad effect produced. For instance, if the gold contained copper there would or might be formed *cuprous oxide* (Cu O) which would cause a red appearance, or there might be *cupric oxide* (Cu O) or copper sulphide (Cu S) both of which would be black. Again, if silver were present *silver sulphide* (Ag S) would be formed and remain as a black insoluble discoloration on the filling, or if iron were present, *Ferric oxide* (Fe O) would be formed, thus giving a red appearance similar to the *cuprous oxide.*

The most feasable theory however is that the discoloration is due to a deposit from the saliva of some insoluble material—*perhaps metal*—for we do not find it in every mouth, even where the same make of gold has been used ; it is only occasionally we find it at all. Sometimes it will be present on one plug, and perhaps the adjoining plug, made at the same sitting, is perfectly free from discoloration of any kind. In this latter case it would seem as if electrical currents had caused its deposition at a particular pole of the minute battery.

We know that forces—chemical and electrical—are at work in nearly every mouth, and at certain times (when there is an acid condition) in all mouths, forces which are capable of destroying and dissolving the strongest enamel, and which will as readily decompose and disintegrate many articles of food, thus setting free their component parts. By this action, iron, copper, lead, and other metals contained in food and medicine, would be deposited not only on the teeth, but on gold plugs. E. C. C.

—*Missouri Dental Journal.*

